# The incidence, pattern and outcome of stray bullet injuries. A growing challenge for surgeons

**DOI:** 10.12669/pjms.295.3794

**Published:** 2013

**Authors:** Arshad M. Malik, Azzam Alkadi, K.Altaf Hussain Talpur, Jawaid Naeem Qureshi

**Affiliations:** 1 Arshad M. Malik, MBBS, FCPS,Associate Professor, Department of Surgery, Liaquat University of Medical & Health Sciences, Jamshoro, Pakistan.; 2 Azzam Alkadi, MD, MSc, FRCSC,Assistant Professor, Department of Surgery,Unaizah College of Medicine, Qassim University, Kingdom of Saudi Arabia.; 3 K.Altaf Hussain Talpur, MBBS, FCPS, Professor, Department of Surgery, Liaquat University of Medical & Health Sciences, Jamshoro, Pakistan.; 4 Jawaid Naeem Qureshi, MBBS, FRCS, Professor, Department of Surgery, Liaquat University of Medical & Health Sciences, Jamshoro, Pakistan.

**Keywords:** Incidental bullet injuries, Morbidity, Mortality, Stray bullet injuries, Young victims

## Abstract

***Objective:*** To study the incidence, pattern of injuries, presentation and management of stray bullet injuries.

***Methods:*** All patients presented and admitted with stray bullet injuries during a period of 4 years from January 2006 to December 2010 were included in this prospective study which was conducted at Liaquat University of Medical and Health Sciences Hospital Hyderabad/Jamshoro. All of the study subjects were admitted through casualty and were initially thoroughly examined and resuscitated. The pattern of injuries was noted and requisite investigations performed. Patients who sustained injuries demanding surgery were prepared accordingly and were submitted for laparotomy or other procedures depending upon the severity of injuries. The data collected on individual basis and variables studied including demographics, pattern of injuries, time since injury occurred and management.

***Results:*** A total number of 165 patients with a mean age of 17.1 years, SD 13.807 and range of 74(2-76) presented with stray bullet injuries during study period. The study population comprised 117(70.90%) males and 48(29.09%) females. Majority of the patients were brought late because of delay in diagnosis or delay in transportation. The commonest victims were young children in their teens and comprised 78% of the study population. Haemothorax/ pneumothorax or peritonitis was the common presentations occurring in 11% and 61.81% of the study population respectively. Of the total number, 92 (55.75%) patients underwent laparotomy while remaining patients either had chest intubation or some other procedures done accordingly. Nine (5.45%) patients developed permanent disabilities while 13(7.87%) patients died either immediately after arrival or later on in the hospital during or after the operative treatment. Mortality was related to the time of arrival in hospital since the injury and thus was highest among those brought 4 or more hours after the shot (P<0.001). Patients who did not sustain major injuries were kept under observation and were subsequently discharged.

***Conclusion:*** Stray bullet injuries are an ever increasing challenge in our society. Unlawful and jubilant use of weapons in celebrations, weddings and similar occasions are causing a lot of morbidity and mortality in the society.

## INTRODUCTION

There is a global increase in unintended fire arm injuries from Europe, to America, UK and Asia.^1^ Public uprisings, protests and aggressive mobs are commonly controlled by using force, and aerial firing is a well known tool used by administrative bodies to disperse the unlawful destructive and aggressive mobs. There are a number of studies published so far indicating gunshot injuries due to weapon violence in the civil society.^2^^-^^5^ Majority of the victims are not even aware that they have sustained gunshot injury.^6^^-^^10^

A number of factors play in the overall incidence of such criminal acts specially in our society such as an undue and inappropriate use of guns, an undue public exposure of the deadly weapons in public meetings and processions by the political workers as well as office bearers of various political parties. There is a tradition of celebrating the marriage ceremonies or the birth of a male child by way of opening aerial fires on such occasions especially in the tribal areas. Stray gun fire celebrations of festivals like kite festival is also claiming many lives every year in Pakistan. This trend had raised the incidence of stray bullet injuries to a substantially high level which has become an open threat to the civil society.

A number of such stray bullet casualties are being reported by youngsters allegedly involved in alcohol abuse. We are facing this problem of stray bullet injuries ever since there is a surge of illegal weapons in the market especially with those who have gangs behind them or some political support. This study was performed to study the incidence, pattern and management of these stray bullet injuries in a major city of Sind province of Pakistan.

## METHODS

During the four years period from January 2006 to December 2010, a total of 165 patients with a history of stray bullet injuries were admitted in a public sector University. There were 117(70.90%) males and 48(29.09%) females with an age range of 2-76 years (Median age, 13 years). An overall 78% of the study population was in the 2-20 years age group. All the patients were admitted through casualty where every patient was managed according to the ALTS guidelines.

The initial assessment was focused on ABC structured physical examination to quickly find out the life threatening conditions. The surgical residents then shift the patients after initial resuscitation. On arrival in the ward, a proforma especially designed for this purpose was attached to the case file of individual patient and all the findings were noted on this by two of the authors. Patient’s characteristics such as symptoms, signs, wound of entry and exit, operative findings, operative and post-operative complications and total number of days of stay in the hospital were noted.

Patients were managed on priority bases after assessment and those who needed surgical intervention were prepared for immediate surgery or any other appropriate procedure accordingly. Patients who were haemodynamically stable were thoroughly investigated by chest x-ray, abdominal x-ray, urine analysis, blood complete picture and serum electrolytes while those who sustained life threatening injuries were immediately resuscitated and managed accordingly. 

Presence of shock and other indications of internal hemorrhage, generalized peritonitis, leakage of intestinal contents or visible intestinal loops were considered strong indications for urgent laparotomy. Bullet injuries to chest with severe dysponea, mediastinal shift, haemo and pneumothorax were considered indications for chest intubation.

The variables studied included demographic details, an account of the mode of injuries, details of the injuries sustained, procedure performed, morbidity and mortality. The results were analyzed statistically on SPSS 17.

## RESULTS

A total of 165 patients regardless of age and gender were admitted in our ward with a history of some sudden unknown injury and were then diagnosed to have sustained a stray bullet injury in a number of different circumstances such as to rejoice victories, aerial firing to disperse the mobs, celebrating firings, injury by a returning bullet while sleeping etc. The demographic details are shown in Table-I. Most of the patients were admitted through casualty presenting with a variety of symptomatology. There was an undue delay in shifting the patients to the hospital due to either unawareness of the bullet injury or because of a delay in the diagnosis while patient was taken to general practitioners who were unable to diagnose the patient and sent the patient back home after prescribing some medicine. Table-II shows the delay between injury and arrival to the hospital in the study subjects. The various modes of presentations are shown in Table-III. A number of different procedures were performed depending upon nature of the injuries as shown in Table-IV.

**Table-I:** Demographics

*Age (in years), Mean * *+* * Standard Deviation (Range) *	*38.14 * *+* * 13.807 (2-76)*	
Age in groups	2-20 years 21-80 years and above	129 (78.18%) 36 (21.81%)
Gender	Males Females	117 (70.90%) 48 (29.09%)

**Table-II:** Time since injury to arrival in Hospital

*Time since injury*	*Frequency*	*Percentage (%)*
Within 1 hour	42	25.45%
2-4 Hours	75	45.45%
4-6 Hours and above	48	29.09%

**Table-III:** Mode of injuries

*Injury *	*Frequency*	*Percentage*
Haemo/Pneumothorax	18	10.90%
Abdominal injury	102	61.81%
Spine injury	17	10.30%
Multiple injuries	20	12.12%
Head injury	8	4.84%
Total	165	99.99%

**Table-IV:** Details of procedures performed

*Procedures Performed*	*Frequency*
Laparotomy+Chest intubation+Fracture and wound management	20
Referal to Neurosurgery	20
Conservative management	15
Laparotomy	92
Chest intubation	18

**Table-V:** Findings on Laparotomy

*Findings*	*Frequency*
Peritoneal penetration	92
No peritoneal penetration	10
Multiple wounds	47
Wound of entry only	43
Wound of entry+ exit	49
Faecal matter coming through wound	17
Sub-cutaneous pallets	12
Intestinal loop visible through wound	09
Distended abdomen with blood ooze through wound	23

**Table-VI:** Pattern of intra-abdominal injuries

*Intra-abdominal injuries*	*Frequency*
Liver	15
Gallbladder	2
Kidneys	4
Small bowel	12
Large bowel	3
Rectum	5
Spleen	2
Multiple injuries	38
Bleeding from mesentry	11

Of the total number, 92 patients sustained bullet injuries to abdomen which demanded immediate laparotomy after initial resuscitation. The criteria for deciding laparotomy included generalized peritonitis, leakage of faecal contents, bowel loops visible through the wound, haemo-peritoneum on abdominal tapping and signs of internal bleeding. Clinical findings on laparotomy in patients with abdominal injuries are shown in Table-V. Pattern of organ injury in abdominal gunshot injuries is shown in Table-VI. Of the total patients treated, nine (5.45%) patients developed permanent disabilities while 13(7.87%) patients died either immediately after arrival or later on in the hospital during or after the operative treatment.

## DISCUSSION

Violence in different forms is an integral part of the routine life in Pakistan. Celebratory gunfire and power show on the streets is increasing dangerously in our society and is an ever growing public health problem that needs a serious consideration. Such unintentional bullet injuries are adding significantly to the morbidity and mortality associated with firearm injuries. A very large number of innocent people are victimized and either loses their lives or rendered handicapped forever. A majority of the victims are not even aware of the incidence and are injured just accidently while passing by a procession or are attending a jubilant gathering celebrating victories, birth of a male child or as a prey to aerial firing to control an aggressive procession. This has resulted in an undue sense of insecurity and fear in people which has restricted their activities and undue exposure to crowds and public places. Children are suffering the most as parents do not let them go out to play in open fields and are only allowed indoor games and supervised sports only. Such deliberate alterations in the life styles are also reported by many authors.^11^^,^^12^

This study was conducted to find out the incidence, pattern and management of such un-intentional bullet injuries. Although more prevalent in our society but it is truly a global problem as indicated by many reports.^13^^-^^15^ We report maximum stray bullet injuries in the age group 2-20 years (78.18%) with a male predominance 971%). This is consistent with the results of many similar studies claiming maximum victims in the similar age group.^16^^-^^21^ Male dominance in our study is also confirmed by many other similar reports .^22^^,^^23^ Jubilation and celebrations on the birth of a male child by aerial firing has become a known accidental killer in tribal and urban areas of Pakistan where it is accepted as a cultural characteristic. The celebratory stray bullet injuries are not unique to Pakistan but a global threat.^24^^,^^25^ This report identifies 7 (4.22%) deaths on such occasions. The incidence of stray bullet injuries in women is surprisingly high in our study and comprises 30% of the total number of study subjects.

Majority of these women were either washing clothes on roofs of their hours or were sleeping at night on roof in open skies when they received a returning bullet into their abdomen or thorax. A number of these women were watching aggressive processions from the gallery and were hit by a stray bullet. The returning bullets causing serious injuries to people deep asleep on roof tops especially in the hot climates is an ever increasing problem in Hyderabad city. Of the total victims in our report, 61.81% had a bullet injury to abdomen leading to laparotomy in majority of them. A New Year night celebration with aerial firing has become a nightmare for innocent bystanders who are not even aware of the nature of such activities.

The major factors behind increasing use of firearm weapons in our society seem to be lack of effective measures by the law enforcing authorities. There is no check and balance on the sale and purchase of ammunition and as a result unauthorized weapons are freely available. A number of states in USA have brought reforms in their legislation for weapon carrying by people and there is a very strict watch on the sale and purchase of such weapons. There is a dire need of such measures to be taken in our country to control this real threat to the mankind.

## CONCLUSION

Stray bullet injuries are becoming a challenging source of morbidity and mortality in our society. Stern actions and legislation are needed to control such unlawful and dangerous celebrations and power show by aerial firings.
